# *FKBP5* genetic variants are associated with respiratory- and sleep-related parameters in Chinese patients with obstructive sleep apnea

**DOI:** 10.3389/fnins.2023.1170889

**Published:** 2023-05-18

**Authors:** Anzhao Wang, Zhicheng Wei, Haolin Yuan, Yaxin Zhu, Yu Peng, Zhenfei Gao, Yuenan Liu, Jinhong Shen, Huajun Xu, Jian Guan, Shankai Yin, Feng Liu, Xinyi Li

**Affiliations:** ^1^Department of Otorhinolaryngology Head and Neck Surgery, Shanghai Sixth People's Hospital Affiliated to Shanghai Jiao Tong University School of Medicine, Shanghai, China; ^2^Shanghai Key Laboratory of Sleep Disordered Breathing, Shanghai, China; ^3^Otorhinolaryngology Institute of Shanghai Jiao Tong University, Shanghai, China

**Keywords:** obstructive sleep apnea, depression, posttraumatic stress disorder, *FKBP5* gene, single nucleotide polymorphism

## Abstract

**Introduction:**

Obstructive sleep apnea (OSA) has been associated with psychiatric disorders, especially depression and posttraumatic stress disorder (PTSD). *FKBP5* genetic variants have been previously reported to confer the risk of depression and PTSD. This study aimed to investigate the association of single nucleotide polymorphisms (SNPs) in the *FKBP5* gene with OSA and OSA-related quantitative traits.

**Methods:**

Four SNPs within the *FKBP5* gene (rs1360780, rs3800373, rs9296158, rs9470080) were genotyped in 5773 participants with anthropometric and polysomnography data. Linear regression and logistic regression analyses were performed to evaluate the relationship between *FKBP5* SNPs and OSA-related traits. Binary logistic regression was used to assess the effect of SNPs on OSA susceptibility. Interacting genes of SNPs were assessed based on the 3DSNP database, and expression quantitative trait loci (eQTL) analysis for SNPs was adopted to examine the correlation of SNPs with gene expression. Gene expression analyses in human brains were performed with the aid of Brain Atlas.

**Results:**

In moderate-to-severe OSA patients, all four SNPs were positively associated with AHI_REM_, and rs9296158 showed the strongest association (*ß* = 1.724, *p* = 0.001). Further stratified analyses showed that in men with moderate OSA, rs1360780, rs3800373 and rs9470080 were positively associated with wake time (*p* = 0.0267, *p* = 0.0254 and *p* = 0.0043, respectively). Rs1360780 and rs3800373 were 28 and 29.4%more likely to rate a higher ordered MAI category (OR (95% CI) = 1.280 (1.042 – 1.575), *p* = 0.019; OR (95% CI) = 1.294 (1.052 – 1.592), *p* = 0.015, respectively). Rs9296158 and rs9470080 increased the risk of low sleep efficiency by 25.7 and 28.1% (OR (95% CI) = 1.257 (1.003 – 1.575), *p* = 0.047; OR (95% CI) = 1.281 (1.026–1.6), *p* = 0.029, respectively). Integrated analysis of eQTL and gene expression patterns revealed that four SNPs may exert their effects by regulating *FKBP5*, *TULP1*, and *ARMC12*.

**Conclusion:**

Single nucleotide polymorphisms in the *FKBP5* gene were associated with sleep respiratory events in moderate-to-severe OSA patients during REM sleep and associated with sleep architecture variables in men with moderate OSA. *FKBP5* variants may be a potential predisposing factor for sleep disorders, especially in REM sleep.

## 1. Introduction

Obstructive sleep apnea (OSA) is one of the most prevalent sleep disorders around the globe, affecting 936 million adults worldwide aged 30–69 years and with a higher incidence in men and older people. China has the largest number of OSA individuals, with approximately 176 million estimated to have an apnea-hypopnea index (AHI) ≥ 5 per hour of sleep (Benjafield et al., 2019). OSA is characterized by recurrent episodes of obstruction in the upper airway during sleep, causing airflow reduction or cessation and resulting in intermittent hypoxia and fragmented sleep, and is usually accompanied by decreased sleep quality, daytime sleepiness and snoring symptoms (Lévy et al., 2015).

Obstructive sleep apnea is often associated with psychiatric disorders, a common comorbidity in patients with mental illness, especially depression and posttraumatic stress disorder (PTSD; Gupta and Simpson, 2015; Krakow et al., 2015; Stubbs et al., 2016; Zhang et al., 2017), both of which are stress-related psychiatric disorders. Depression exhibits negative alterations in mood, sleep, appetite, psychomotor activity and cognition (Dwyer et al., 2020). PTSD is characterized by intrusion, avoidance, cognitive and mood changes, and alterations in arousal after trauma events (Jaoude et al., 2015). Several studies have claimed a bidirectional and complicated connection linking OSA and psychiatric disorders (Gupta and Simpson, 2015; Jaoude et al., 2015). It has been reported that OSA prevalence is increased in individuals suffering from depression or PTSD (Hattori et al., 2009), and OSA patients are at higher risk of psychiatric comorbid conditions, including depression, PTSD, anxiety disorder or psychosis (Sharafkhaneh et al., 2005). Both OSA and depression/PTSD are reported to share overlapping symptoms, including sleep disturbances, negative changes in cognitions and mood, and a lower quality of life. Psychiatric symptoms such as nightmare, anxiety and depressive state in PTSD or depression patients were also significantly relieved after CPAP treatment for comorbid OSA, suggesting that a potential complex interplay may exist between the two disorders (Habukawa et al., 2010; Tamanna et al., 2014). Researchers have suggested that multiple factors may play a role in the association of depression and PTSD with OSA because they have similarities in disrupted biological pathways, including neurotransmitter imbalances, hypothalamus-pituitary–adrenal (HPA) axis disturbances, dysregulated inflammatory pathways, and increased oxidative stress (Gozal et al., 2008; Lopresti and Drummond, 2013; Tauman et al., 2014). However, potential clues regarding genetic links remain unclear, although genetic factors contribute not only to psychiatric disorder development but also to OSA etiology and phenotype (Colvonen et al., 2018; Xu et al., 2022). Potential genetic background overlap between stress-related psychiatric disorders and OSA has yet to be explored.

Glucocorticoids (GCs) help control the circadian sleep–wake rhythm and maintain sleep homeostasis, and prolonged exposure to GCs can lead to sleep alterations (Nicolaides et al., 2000). *FKBP5* is a glucocorticoid-responsive gene and is also essential for the negative feedback of the HPA axis (Hähle et al., 2019), indicating that *FKBP5* may also exert an effect on sleep modulation. The *FKBP5* gene has emerged as one of the most promising and comprehensively studied candidate genes for PTSD and depression thus far (Smoller, 2016). A large body of research has found that single nucleotide polymorphisms (SNPs) in *FKBP5* are associated with a number of psychiatric disorders, including mood and anxiety disorders, PTSD, psychosis, and substance abuse disorders (Binder et al., 2008; Xie et al., 2010; Zimmermann et al., 2011; Klengel et al., 2013). Those findings mainly focused on depression and PTSD, reporting that *FKBP5* variations are also associated with psychiatric-related phenotypes, including post-traumatic pain severity (Bortsov et al., 2013), and with recurrence of depressive episodes and response to antidepressant treatment (Binder et al., 2004), which has been widely observed and validated in patients across different races (Binder, 2009; Zannas and Binder, 2014; Matosin et al., 2018). In individuals with higher induction alleles, impaired negative feedback of the HPA axis altered by *FKBP5* variants results in a prolongation of stress hormone system activation under stress exposure. Among these genotypes, the most widely studied four SNPs, namely, rs1360780, rs3800373, rs9296158, and rs9470080, contribute to the risk and disease severity of depression and PTSD (Binder et al., 2004, 2008; Xie et al., 2010; Zimmermann et al., 2011), so we chose the four variants as our research object.

Concerning the high susceptibility to OSA in mental disorders and the role of *FKBP5* in glucocorticoid action, it is plausible to hypothesize that genetic variants of *FKBP5*, which is a PTSD and depression candidate gene, may have an impact on OSA. To date, no study has investigated the association between OSA and PTSD/depression at the genetic level. Given the lack of data implicating the influence of *FKBP5* variants on sleep regulation, we conducted a large cross-sectional study to evaluate the association of *FKBP5* polymorphisms with OSA and OSA-related quantitative clinical traits in a Chinese Han OSA population.

## 2. Methods

### 2.1. Participants

Subjects suspected of having OSA were enrolled from the Shanghai Sleep Health Study (SSHS) cohort, in which people were hospitalized and observed in the sleep center of Shanghai Sixth People’s Hospital Affiliated to Shanghai Jiao Tong University School of Medicine. In addition to the genomic study, demographic information and clinical measurement data were also collected for each individual, from whom written informed consent was obtained (Xu et al., 2022). Next, subjects from SSHS were screened according to the following exclusion criteria. (i) age less than 18 years old; (ii) history of continuous positive airway pressure (CPAP) treatment, oral appliance therapy or upper airway surgery; (iii) history of other sleep disorders (i.e., narcolepsy, restless leg syndrome, or upper airway resistance syndrome); (iv) systemic disease (i.e., chronic liver disease, chronic kidney disease), cancer, hyperparathyroidism, or hypoparathyroidism; (v) cardiopulmonary diseases. (vi) unavailable standard polysomnography data. A total of 5,773 participants were ultimately analyzed in this study. All cases and controls were of Han Chinese descent and mainly from eastern China.

### 2.2. Anthropometric measurements and Epworth sleepiness scale questionnaire

Anthropometric parameters, including height (m) and weight (kg), were measured in barefoot subjects wearing lightweight clothing. Waist circumference (WC, cm) was measured in the middle of the lowest rib margin and iliac crest during gentle exhalation. Hip circumference (HC, cm) was defined as the maximum circumference over the buttocks at the baseline visit. The body mass index (BMI, kg/m^2^) was calculated as weight divided by height squared, and waist–hip ratio (WHR) was calculated as WC divided by HC.

The effect of sleep apnea on excessive daytime sleepiness was assessed using the verified Chinese version of the Epworth Sleepiness Scale (ESS; Johns, 1991). All participants were asked to complete a self-administered questionnaire evaluating their subjective daytime sleepiness level by eight items, with overall scores ranging from 0 to 24 and higher scores indicating more daytime sleepiness (Johns, 1991).

### 2.3. Polysomnography and definition of quantitative phenotypic variables

All participants underwent nocturnal polysomnography (PSG) monitoring (Alice 4 or 5; Respironics, Pittsburgh, PA, United States) in the sleep center. Data containing respiratory events, oxygen traits and objective sleep-related variables that were recorded by polysomnographic device were then manually scored and checked by experienced sleep technicians according to the 2012 criteria of the American Academy of Sleep Medicine (AASM; Berry et al., 2012).

Apnea was defined as a reduction in oronasal airflow from baseline by ≥ 90% for at least 10 s. Hypopnea was defined as a drop in signal excursions from baseline by ≥ 30% for ≥ 10 s, accompanied by a decrease in oxygen desaturation of ≥ 3% or an associated arousal from sleep. The apnea–hypopnea index (AHI) was calculated based on the mean number of apnea and hypopnea events per hour of sleep. OSA severity was quantified by AHI, and non-OSA, mild, moderate, and severe OSA were defined as AHI < 5.0, 5.0–14.9, 15.0–29.9 and ≥ 30.0, respectively. AHI_REM_ and AHI_NREM_ were calculated as the numbers of AHI per hour of REM and non-REM (NREM) sleep.

The oxygen desaturation index (ODI) was defined as the total number of episodes of ≥ 3% arterial oxygen desaturation per hour of sleep. LSpO2 referred to the lowest oxyhemoglobin saturation.

Sleep period time (SPT) was defined as the time from the first epoch of sleep to final awakening, and total sleep time (TST) was defined as the total time in any stage of sleep. Sleep efficiency (SE) was calculated as TST/TIB (time in bed). Wake time (WK) during sleep was defined as the total time awake during SPT in our study when electroencephalogram (EEG) showed predominantly α rhythm activity on eye closure during quiet alertness, or eye blinking and eye movement with normal or high chin muscle tone detected by electrooculogram and electromyogram. Sleep time comprised non-rapid eye movement (NREM) sleep and rapid eye movement (REM) sleep, with the former categorized into stage N1, N2 and N3. Stage N1 represents sleep onset, in which α waves are attenuated and replaced by mostly θ waves. Stage N2 was characterized by “K-complexes” and “sleep spindles,” and stage N3 reflected slow wave sleep. Stage REM or R was defined when rapid eye movements, low-voltage mixed-frequency brain wave activity and low chin muscle tone were present. The ratios were also calculated as follows: Wake time during SPT (WK/SPT%); REM during SPT (REM/SPT%); REM during TST (REM/TST%); N1, N2, and N3 sleep times during SPT (N1, N2, and N3/SPT%); and N1, N2, and N3 sleep times during TST (N1, N2, and N3/TST%). Arousal was defined as an abrupt shift in the EEG frequency, including α, θ and/or other frequencies ≥16 Hz (but not spindles), lasting for ≥ 3 s. The microarousal index (MAI) referred to the mean number of arousals per hour of sleep.

### 2.4. Single nucleotide polymorphism selection and genotyping

Four SNPs in the FKBP5 gene that have previously been reported to be associated with both PTSD and depression (rs1360780, rs3800373, rs9296158, and rs9470080) were enrolled in this study (Binder et al., 2004, 2008; Xie et al., 2010; Zimmermann et al., 2011). Genomic DNA was extracted from blood samples of each participant by a DNA isolation kit (Qiagen). Affymetrix Axiom Genome-Wide CHB Array (CHB) was used to detect the specific base sequence, and information about these 4 SNPs for every participant was obtained from this genomic database. The call rates of rs1360780, rs3800373, rs9296158, and rs9470080 were 100, 97.7, 99.1 and 100%, respectively. All selected *FKBP5* SNPs met Hardy–Weinberg equilibrium in cases and controls (*p >* 0.05), with minor allele frequencies >0.01.

### 2.5. Statistical analysis

Statistical analyses were performed using SPSS 26.0 software (IBM Corp., Armonk, NY, United States). The normally distributed data are presented as the means and standard deviation; skewed data are presented as the median (IQR), and categorical data are presented as the number (percentage). Differences in the baseline characteristics among the four groups were examined using one-way analysis of variance (ANOVA), non-parametric Kruskal–Wallis *H* test, or *χ*^2^ tests according to the type of data distribution. The Hardy–Weinberg equilibrium test was assessed using PLINK (v1.90) before association analysis (Purcell et al., 2007). Linkage disequilibrium analysis was performed by Haploview (version 4.2) by calculating |D′| and *r*^2^, and the haplotype block structure was estimated by the confidence interval (CI) algorithm (Gabriel et al., 2002; Barrett et al., 2005). OSA-related quantitative phenotypic variables were analyzed under the additive genetic model by linear regression. Logistic regression analyses were performed to evaluate the relationship between polymorphisms and categorical variables. For AHI_REM_ or microarousal index analysis, all subjects were grouped into 4 ordered categories by AHI_REM_ or MAI quartiles (Qs). Odds ratios (ORs) and 95% confidence intervals (CIs) were estimated by multinomial or ordinal logistic regression, using patients in the first quartile (Q1) of AHI_REM_ or MAI as the reference category. The ordinal logistic model assumed that these odds ratios were equal between the adjacent ordered categorical dependent variables and was adopted under the proportional odds model, which was verified by parallel line testing (*p* > 0.05; Guan et al., 2016). If the proportional odds assumption was not met by the test of parallel lines (*p* < 0.05), we performed multinomial logistic regression with unordered categorical variables instead. Logistic regression analyses were also used to assess the effect of SNPs on the risk of OSA. A two-tailed *p* value < 0.05 was considered statistically significant.

### 2.6. Expression quantitative trait loci and gene expression analysis

Expression quantitative trait loci (eQTL) analysis of SNPs was obtained from Ensembl data.^1^ Data from the 3DSNP database were used to investigate the link between SNPs and their three-dimensional interacting genes through 3D chromatin loops.^2^ Human brain normalized microarray expression data were downloaded from the Allen Human Brain Atlas,^3^ and then a heatmap was drawn displaying relevant gene expression levels in different parts of the human brain by GraphPad Prism software (GraphPad Prism v9.0.0).

## 3. Results

### 3.1. Baseline characteristics

In the current study, we enrolled a total of 5,773 individuals of Han Chinese ancestry, comprising 5,029 OSA patients (314 mild OSA, 1199 moderate OSA, 3516 severe OSA) and 744 non-OSA controls. The baseline characteristics of the participants in the four groups are shown in Table 1. Age, the proportion of male patients, BMI, AHI_REM_ and AHI_NREM_, microarousal index, percentages of N1 time/TST, ODI and daytime sleepiness were positively correlated with increasing OSA severity. Meanwhile, the percentages of REM/TST, N3/TST and lowest oxyhemoglobin saturation decreased with increasing OSA severity.

**Table 1:** Basic characteristics of the overall population classified by the severity of obstructive sleep apnea (OSA).

Traits	Non-OSA (744)	Mild OSA (314)	Moderate OSA (1199)	Severe OSA (3516)	*p* Value
Male (%)	100.00%	99.00%	79.23%	88.14%	<0.001
Age	35 (29–45)	33 (29–36)	44 (35–55)	43 (35–53)	<0.001
BMI (kg/m2)	24.2 (22.4–26.2)	25.2 (23.3–27.1)	26.1 (24.2–28.4)	27.7 (25.6–30.1)	<0.001
WHR	0.91 (0.85–0.97)	0.92 (0.87–0.97)	0.94 (0.88–1)	0.96 (0.93–0.99)	<0.001
AHI/total	2.1 (0.9–3.4)	10.8 (8.8–13.0)	22.2 (18.3–25.9)	57.1 (44.2–70.1)	<0.001
AHI_REM_	1.9 (0–5.2)	16.7 (7.4–27.1)	31.1 (15.7–45.2)	56.4 (43.6–67.5)	<0.001
AHI_NREM_	1.9 (0.8–3.3)	9.8 (7.7–12.3)	20.7 (17.0–25.2)	56.2 (41.7–70.6)	<0.001
REM (min)	43.0 (24.0–63.0)	45.5 (24.5–66.0)	42.5 (24.8–62.0)	41.5 (24.5–60.9)	0.145
REM/SPT (%)	10.4 (5.9–14.9)	10.6 (5.8–15.4)	9.9 (5.9–14.2)	9.6 (5.8–13.8)	0.027
REM/TST (%)	11.7 (6.8–16.2)	11.6 (6.7–17.1)	10.9 (6.5–15.7)	10.4 (6.5–14.7)	<0.001
MAI	14.4 (10–22.6)	17.9 (12.5–26.8)	21.2 (13.8–30.8)	35.4 (20.2–53.8)	<0.001
WK (min)	22 (3.3–61.5)	17.1 (1.0–50.5)	20.0 (3.0–54.7)	18.7 (4.0–49.0)	0.094
WK/SPT (%)	5.5 (0.8–14.2)	4.2 (0.2–11.2)	4.8 (0.8–13.0)	4.3 (1.0–11.1)	0.016
N1 (min)	62.0 (38.8–100.3)	62.8 (37.5–98.0)	72 (45–110.8)	74.5 (44.0–118.5)	<0.001
N1/SPT (%)	15.6 (10.2–24.9)	15.5 (10.0–24.8)	16.6 (10.2–26.5)	17.1 (10.2–26.8)	0.094
N1/TST (%)	17.0 (10.7–27.7)	16.8 (10.4–24.8)	18.8 (11.1–29.4)	19.1 (11.3–29.7)	0.001
N2/(min)	189 (131.5–237.0)	201.8 (150.0–248.5)	194 (139.5–244.5)	210.5 (152.5–262.5)	<0.001
N2/SPT (%)	45.0 (33.0–54.3)	46.4 (36.2–55.8)	45.2 (34.2–55.4)	48.5 (35.9–58.4)	<0.001
N2/TST (%)	50.4 (40.0–59.0)	51.5 (42.6–59.6)	50.7 (40.1–59.5)	53.1 (41.2–62.4)	<0.001
N3/(min)	64.5 (39.0–103.4)	62.8 (39.0–86.9)	59.0 (34.5–94.5)	49.0 (22.0–90.1)	<0.001
N3/SPT (%)	15.7 (9.7–24.7)	14.7 (9.3–20.8)	14.1 (8.2–22.0)	11.4 (5.7–21.0)	<0.001
N3/TST (%)	15.7 (9.7–24.7)	16.3 (10.5–22.9)	15.5 (9.1–24.1)	12.1 (5.9–22.5)	<0.001
TST (min)	396.1 (337.5–436.4)	405.4 (352.5–440.0)	405.5 (349.6–444.6)	418.2 (367.3–455.3)	<0.001
Sleep efficiency	86.2 (74.6–94.0)	86.0 (76.7–93.2)	87.0 (77.5–94.7)	90.9 (81.9–96.2)	<0.001
LSpO2	92 (90–95)	87 (84–90)	83 (78–87)	71 (62–79)	<0.001
ODI	2.3 (1.1–3.9)	10.7 (7.9–13.5)	22.3 (17.7–27.8)	57.9 (43.8–72.2)	<0.001
ESS	5 (1–10)	7 (4–11)	7 (3–11)	10 (5–14)	<0.001

The normally distributed data are presented as the means and standard deviation; skewed data are presented as the median (IQR), and categorical data are presented as the number (percentage). Differences in the baseline characteristics among the four groups were examined using ANOVA, non-parametric Kruskal–Wallis test and Chi-square test according to the type of data.

AHI, apnea–hypopnea index; BMI, body mass index; WHR, waist/hip ratio; REM, rapid eye movement; NREM, non-rapid eye movement; MAI, microarousal index; WK, wake time; N1, stage 1 sleep of non-rapid eye movement; N2, stage 2 sleep of non-rapid eye movement; N3, stage 3 sleep of non-rapid eye movement; TIB, time in bed; SPT, sleep period time; WK/SPT (%), percentage of wake time during sleep period time; TST, total sleep time; Sleep efficiency TST/TIB, LSpO2, lowest oxyhemoglobin saturation; ODI, oxygen desaturation index; ESS, Epworth sleepiness scale.

The basic characteristics of SNPs within the *FKBP5* gene are listed in Table 2. The genotype frequencies of all variants were in Hardy–Weinberg equilibrium (Supplementary Figure S1). The minor allele frequencies of rs3800373 and rs1360780 between the non-OSA and OSA groups were significantly different (*p* = 0.009 and *p* = 0.016, respectively).

**Table 2:** Information on each selected SNP.

SNP	Position	Functional consequence	Minor/major allele	Risk allele	MAF	*p* allele
rs3800373	35,542,476	3′-UTR	C/A	C	0.2575	0.009
rs9296158	35,567,082	Intron	A/G	A	0.3208	0.160
rs1360780	35,607,571	Intron	T/C	T	0.2599	0.016
rs9470080	35,646,435	Intron	T/C	T	0.3273	0.121

MAF, minor allele frequency.

*p* minor allele distribution between cases and controls from Chi-squared test.

All the selected SNPs are located within the *FKBP5* gene region on chromosome 6.

### 3.2. Associations of *FKBP5* SNPs with respiratory events

**Figure 1:**
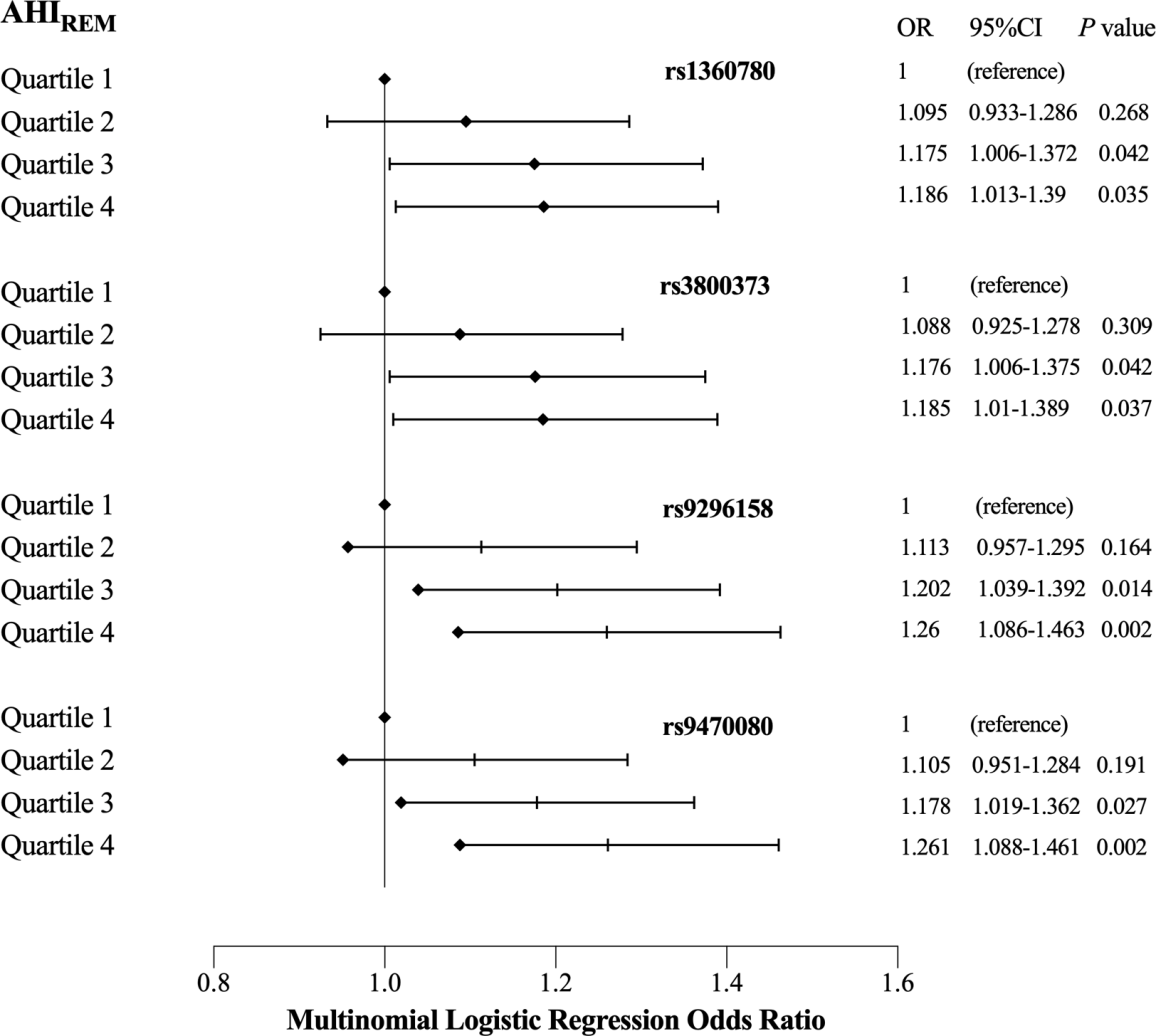
Effects of single nucleotide polymorphisms (SNPs) and quartiles on each quartile of AHI_REM_ in moderate to severe obstructive sleep apnea (OSA) patients (reference = Quartile1). OR (odds ratio) and 95% CI were obtained by multinomial logistic regression using the first quartile of AHI_REM_ as the reference category, and regression models were adjusted for age, sex and BMI. Diamonds indicate ORs, and horizontal dark lines indicate 95% CIs.

We investigated the association between the four psychiatric disorder-associated SNPs in the *FKBP5* gene and OSA-related quantitative sleep parameters, including respiratory events, oxygen traits and objective sleep-related variables. We observed no significant association between the SNPs and quantitative traits across all the participants and the non-OSA group, which are listed in the Supplementary Tables. However, in moderate-to-severe OSA patients (AHI ≥ 15, *n* = 4,715), linear regression analysis revealed that all SNPs were positively associated with AHI_REM_, and rs9296158 showed the strongest association with AHI_REM_ (*ß* = 1.724, *p* = 0.001), even after adjusting for age, sex and BMI (Table 3). We then stratified AHI_REM_ into quartiles corresponding to an AHI_REM_ of Q1 (AHI_REM_ < 20.3), Q2 (20.3 ≤ AHI_REM_ < 44.9), Q3 (44.9 ≤ AHI_REM_  < 60.9), and Q4 (AHI_REM_ ≥ 60.9). Multinomial logistic regression analyses were employed to evaluate the impacts of SNPs on each quartile of AHI_REM_ using Q1 as the reference category (Figure 1), which showed that all four SNPs increased the likelihood of belonging to higher quartiles of AHI_REM_. Rs9296158 or rs947000 carrying the psychiatric disorder-related risk A or T allele was also related to a graded increase in the risk of having the highest quartile (Q4) of AHI_REM_ compared to the reference category (Q1) in the moderate-to-severe OSA population (OR (95% CI) = 1.26 (1.086–1.463), *p* = 0.002; OR (95% CI) = 1.261 (1.088–1.461), *p* = 0.002, respectively). There were no associations of SNPs with oxygen traits.

**Table 3:** Association of SNPs with clinical features related to respiratory events in moderate-to-severe OSA patients (*n* = 4,715).

Traits	SNP	AA	Aa	aa	*β*	*p*	*β* ^*^	p^*^
AHI _REM_	rs1360780	47.27 ± 23.12	48.25 ± 23.03	49.92 ± 21.80	1.160	**0.034**	1.184	**0.027**
AHI _REM_	rs3800373	47.28 ± 23.15	48.22 ± 22.92	49.74 ± 22.01	1.088	**0.049**	1.195	**0.026**
AHI _REM_	rs9296158	46.96 ± 23.19	48.02 ± 23.03	50.97 ± 21.82	1.663	**0.001**	1.724	**0.001**
AHI _REM_	rs9470080	46.87 ± 23.15	48.17 ± 23.04	50.46 ± 22.08	1.626	**0.002**	1.7	**0.001**
AHI _REM_	haplotype	47.23 ± 23.18	47.95 ± 22.94	49.85 ± 22.24	1.113	**0.028**	1.194	**0.016**
AHI_NREM_	rs1360780	47.24 ± 23.30	47.33 ± 23.36	48.48 ± 22.57	0.373	0.500	0.627	0.242
AHI_NREM_	rs3800373	47.29 ± 23.23	47.25 ± 23.31	48.48 ± 22.60	0.295	0.595	0.607	0.261
AHI_NREM_	rs9296158	47.40 ± 23.13	47.09 ± 23.39	48.29 ± 22.90	0.175	0.737	0.448	0.374
AHI_NREM_	rs9470080	47.38 ± 23.05	47.12 ± 23.41	48.20 ± 23.17	0.186	0.743	0.438	0.381
AHI_NREM_	haplotype	47.33 ± 23.19	48.17 ± 23.36	48.00 ± 22.93	0.167	0.743	0.463	0.350

AA represents the homozygotes of the major allele, Aa represents the heterozygotes and aa represents the homozygotes of the minor allele.

*p* values were calculated by using linear regression models, and p^*^ were adjusted for age, sex, and BMI. Data are shown as the mean ± SD. *p* values < 0.05 are shown in bold.

### 3.3. Associations of *FKBP5* SNPs with sleep-related variables

**Figure 2:**
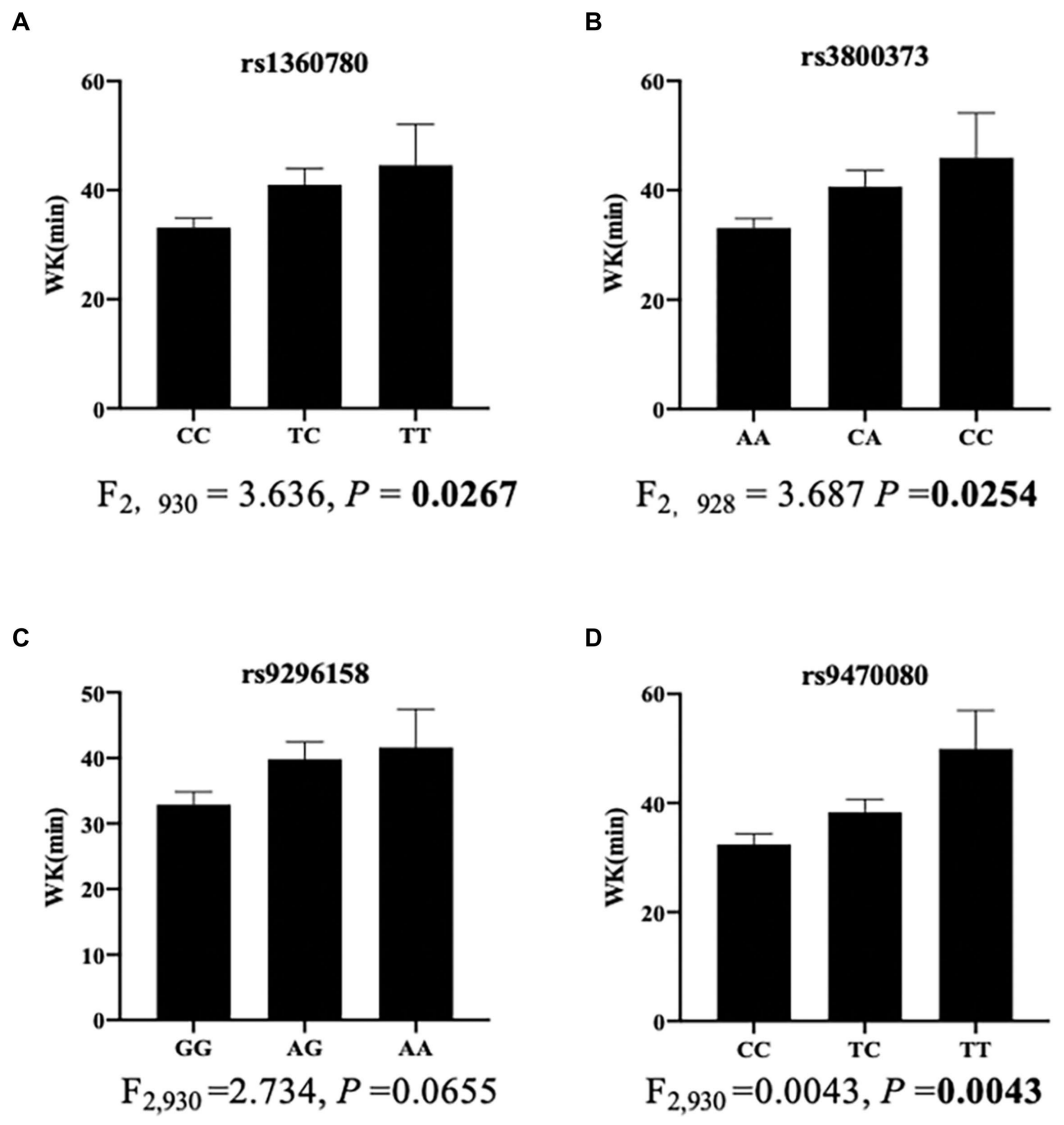
Association of SNPs with wake time during sleep period time in men with moderate OSA (*n* = 950). The bar charts showed the associations of rs1360780 **(A)**, rs3800373 **(B)**, rs9296158 **(C)** and rs9470080 **(D)** with wake time. Data are shown as the means ± SEMs. The *p* value was calculated using ANOVA.

Further stratified analyses showed that in male moderate OSA subjects, *FKBP5* SNPs rs1360780, rs3800373 and rs9470080 were positively associated with WK during SPT (*p* = 0.0267, *p* = 0.0254 and *p* = 0.0043, respectively; Figure 2). Rs1360780, rs3800373 and rs9470080 showed significant positive associations with percentage of wake time during sleep period time (WK/SPT%) after adjusting for age and BMI (all *p* < 0.05; Table 4). For microarousal, all subjects were grouped into 4 ordered categories by MAI quartile, and we adopted an ordinal logistic regression analysis (proportional odds assumption verified by parallel line test) to estimate the effects of SNPs on MAI (Table 5). The results showed that both rs1360780 and rs3800373 were 28 and 29.4%more likely to rate a higher ordered MAI category, which means that these two SNPs were positively associated with the severity of sleep fragmentation after adjustment for age and BMI (OR (95% CI) = 1.280 (1.042–1.575), *p* = 0.019; OR (95% CI) = 1.294 (1.052–1.592), *p* = 0.015, respectively). Moreover, rs9296158 and rs9470080 increased the risk of low sleep efficiency (TST/TIB ≤ 80%) by 25.7 and 28.1% (OR (95% CI) = 1.257(1.003–1.575), *p* = 0.047; OR (95% CI) = 1.281(1.026–1.6), *p* = 0.029, respectively; Table 6). No association was detected between *FKBP5* genetic variants and the risk of moderate OSA (*p* > 0.05; Supplementary Table S4).

**Table 4:** Association of SNPs with sleep-related traits in men with moderate OSA (*n* = 950).

Traits	rs3800373				rs9296158				rs1360780				rs9470080			
	*β*	*p*	β^*^	p^*^	*β*	*p*	β^*^	p^*^	*β*	*p*	β^*^	p^*^	*β*	*p*	β^*^	p^*^
MAI	1.348	0.172	1.529	0.129	1.451	0.142	1.645	0.102	0.314	0.734	0.117	0.687	0.809	0.371	0.885	0.338
MAI (4 categories)	0.112	**0.038**	0.130	**0.018**	0.116	**0.031**	0.135	**0.015**	0.058	0.25	0.069	0.178	0.082	0.096	0.091	0.071
WK (SPT) (min)	6.873	**0.008**	6.444	**0.016**	7.033	**0.007**	6.574	**0.014**	5.424	**0.026**	4.521	0.070	7.659	**0.001**	6.985	**0.004**
WK/SPT (%)	1.354	**0.02**	1.240	**0.039**	1.366	**0.02**	1.246	**0.039**	1.017	0.062	0.808	0.150	1.444	**0.007**	1.275	**0.021**
WK (TIB) (min)	5.833	0.16	5.174	0.230	6.738	0.105	6.064	0.160	7.880	**0.043**	7.115	0.079	10.953	**0.004**	10.578	**0.008**
WK (TIB)/TIB (%)	0.778	0.31	0.610	0.444	0.998	0.193	0.831	0.297	1.209	0.092	1.031	0.168	1.565	**0.026**	1.435	0.050
TST/TIB	−0.665	0.373	−0.463	0.549	−0.757	0.313	−0.548	0.480	−0.858	0.219	−0.615	0.395	−1.2	0.08	−0.984	0.167
TST/TIB (2 categories)	0.038	0.05	0.031	0.214	0.037	0.132	0.03	0.234	0.054	**0.017**	0.047	**0.046**	0.056	**0.013**	0.050	**0.029**

β^*^ and p^*^ were adjusted for age and BMI when multiple linear regression models were adopted.

**Table 5:** Effects of SNPs on microarousal index in men with moderate OSA (*n* = 950).

SNP	OR	95% CI	*p*	OR^*^	95% CI^*^	*p* ^*^
rs1360780	1.237	(1.013-1.513)	**0.037**	1.280	(1.042–1.575)	**0.019**
rs3800373	1.249	(1.022–1.527)	**0.030**	1.294	(1.052–1.592)	**0.015**
rs9296158	1.122	(0.931–1.354)	0.226	1.150	(0.947–1.395)	0.157
rs9470080	1.171	(0.976–1.408)	0.091	1.195	(0.988–1.443)	0.066
haplotype	1.022	(1.001–1.045)	**0.042**	1.025	(1.003–1.048)	**0.025**

The ORs with 95% CIs are shown for the minor allele.

The microarousal index was classified into four ordered categories by quartile, and ordinal logistic regression was performed using a proportional odds model that passed the parallel lines test (*p* > 0.05).

* Adjusted for age and BMI.

**Table 6:** Association of the SNPs with sleep efficiency in men with moderate OSA (*n* = 950).

	OR	95% CI	*p*	OR^*^	95% CI^*^	*p* ^*^
rs1360780	1.201	(0.952-1.514)	0.123	1.164	(0.916–1.480)	0.215
rs3800373	1.196	(0.947–1.510)	0.132	1.157	(0.909–1.473)	0.235
rs9296158	1.298	(1.044–1.615)	**0.019**	1.257	(1.003–1.575)	**0.047**
rs9470080	1.313	(1.059–1.627)	**0.013**	1.281	(1.026–1.600)	**0.029**
haplotype	1.184	(0.959–1.462)	0.117	1.152	(0.926–1.434)	0.205

Sleep efficiency TST/TIB, total sleep time/time in bed.

Sleep efficiency dichotomized into bad (1, SE ≤ 80%) vs. > good (0, SE > 80%).

* Adjusted for age and BMI.

### 3.4. Integrated analysis revealed *FKBP5* SNP-affected genes in sleep-breathing regulation

We assessed the relationship between the four SNPs and their interacting genes. Based on data from the 3DSNP database, rs1360780 interacted with six protein-coding genes (*ARMC12*, *CLPS*, *CLPSL1*, *CLPSL2*, *FKBP5*, *TULP1*) in various tissues, and the other 3 SNPs were linked to five genes via chromatin loops (Supplementary Table S5). Among the SNP-interacting genes, *TULP1* is located ~ 60 kb downstream from the *FKBP5* gene, while *ARMC12* is located ~ 3 kb upstream from the *FKBP5* gene (Supplementary Figure S2). We examined the expression quantitative trait loci (eQTL) information from the Ensembl database to investigate whether these SNPs were correlated with the expression of nearby genes, by which these variants might exert their functions in different human tissues. These data suggested that the four SNPs were also all associated with several genes, including *TULP1*, *ARMC12*, *ETV7*, *SRPK1, PXT1*, *BRPF3*, *RPL10A*, *STK38*, and *FKBP5,* in multiple tissues. Among them, all four SNPs were correlated with the expression of *TULP1* in multiple tissues, showing the strongest correlation, and rs1360780 was associated with a marked decrease in *TULP1* expression in the heart atrial appendage (*p* = 8.93 × 10^−13^). Rs3800373 was shown to be associated with increased expression of *ARMC12* in the muscular layer of the esophagus (*p* = 6.95 × 10^−5^), with the second strongest correlation (Supplementary Table S6). In addition, the four SNPs were also associated with *FKBP5* gene expression changes in various tissues, including brain tissues (Supplementary Table S7). For instance, rs9470080 was associated with a significant increase in *FKBP5* expression in induced pluripotent stem cells (*p* = 0.004).

**Figure 3:**
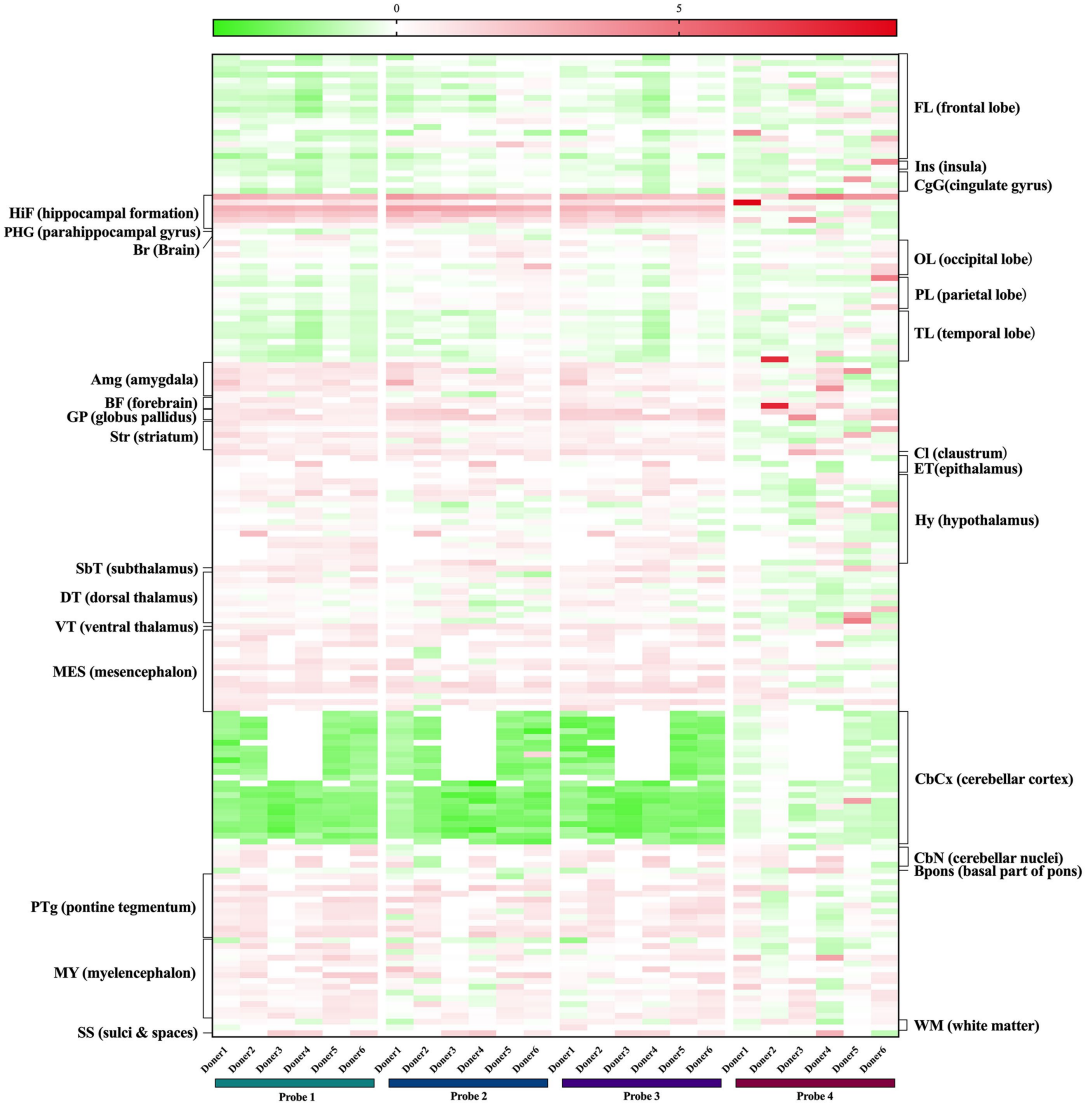
Expression of *FKBP*5 in human brains from the Allen Brain Atlas. *Probe1* A_23_P111206, *Probe2* A_24_P38081, *Probe3* CUST_1369_PI416573500, *Probe4* CUST_1412_PI416379584.

Moreover, we also examined human brain normalized expression data of the aforementioned genes from the Allen Human Brain Atlas. Microarray data of six donors using four *FKBP5* gene probes revealed that *FKBP5* was predominantly expressed in the hippocampus and amygdala, which was consistent with previous literature showing that *FKBP5* regulates the stress response mainly in the hippocampus and amygdala of the human and mouse brain. In addition, *FKBP5* was expressed moderately in various neural pontine tegmentum nuclei and medullary nuclei (Figure 3), and neural circuits in the pons and medulla are known to control breathing during sleep, with the pontine tegmentum involved in the generation and maintenance of REM sleep (Cox et al., 2016). *FKBP5* was also moderately expressed in the striatum, globus pallidus and ventral tegmental area located in the midbrain, all of which play a key role in wakefulness control (Eban-Rothschild et al., 2016; Luo et al., 2018; Yu et al., 2019; Dong et al., 2022). The expression of *TULP1* was detected in several neurons in the hippocampus, subthalamus, midbrain and medulla, while *ARMC12* expression was detected in some neurons in the striatum, midbrain and medulla (Supplementary Figure S3), indicating that *TULP1* and *ARMC12* may have potential roles in the regulation of sleep structure likewise. Taken together, these results suggested that the four SNPs may exert their function in sleep breathing regulation by affecting *FKBP5*, *TULP1* and *ARMC12*. However, this speculation still requires further confirmation from experiments to determine whether these genes play a part in the regulation of breathing in REM sleep and wakefulness.

## 4. Discussion

In the current study, we identified the association between *FKBP5* SNPs and quantitative phenotypic variables in a large-scale Chinese OSA cohort study. Rs1360780, rs3800373, rs9296158, and rs9470080 are all positively associated with AHI_REM_, which suggests that carriers of psychiatric-related risk alleles are prone to suffering from sleep breathing events, especially in the REM sleep stage in OSA patients. *FKBP5* variants potentially contribute to sleep breathing disorders.

Another finding in our study regarding sleep architecture showed that three of the four SNPs (rs1360780, rs3800373 and rs9470080) were positively associated with wake time. In addition, two SNPs (rs1360780 and rs3800373) were associated with the microarousal index, and the other two (rs9296158 and rs9470080) increased the risk of lower sleep efficiency in the male moderate OSA group. Considering that wake time during sleep period time and microarousal are microscopic indicators of sleep fragmentation, our results therefore suggested that *FKBP5* SNPs may promote sleep fragmentation in moderate OSA. However, these findings were not observed in the severe OSA group, possibly because factors including age and BMI are more important risk factors than genetic variants for sleep architecture in severe OSA, and therefore, the correlation of *FKBP5* variants with sleep parameters is not strong enough for the prediction of regression models. Additionally, the sample size of female participants diagnosed with moderate OSA might be too small to blame for the irrelevant findings in their female counterparts.

Posttraumatic stress disorder, depression and OSA share sleep disturbance as a common typical feature. Several studies have performed polysomnographic recording to examine the sleep patterns in PTSD and depression (Lavie, 2001; van Liempt et al., 2013; Baglioni et al., 2016; Zhang et al., 2019), which were characterized by a highly aroused state and fragmented sleep. Specific pathophysiological mechanisms underlying the relationship between psychiatric disorders and OSA remain unknown. Krakow et al. (2015) proposed a bidirectional pathway theory model assuming that nightmares and arousals occurring in PTSD result in sleep fragmentation concomitantly, leading to the development of OSA, which may in turn worsen PTSD symptoms. Sleep fragmentation significantly decreases pharyngeal critical closing pressure (Pcrit) corresponding to increased airway collapsibility (Sériès et al., 1994), leading to more sleep breathing events and higher susceptibility to OSA, predominantly in the REM stage. Obstructive respiratory events occur more frequently and with longer duration during REM sleep than NREM sleep in OSA due to increased airway collapsibility caused by reduced pharyngeal muscle response and respiratory effort throughout REM sleep (Findley et al., 1985; Shea et al., 1999). Coincidently, our finding that *FKBP5* variants were associated with respiratory events only during REM sleep suggested a common genetic background underpinning the comorbidity of OSA and PTSD/depression. However, whether *FKBP5* SNPs could function through Krakow’s model as common genetic factors still requires further functional verification. In addition, Tseng et al. (2022) recently demonstrated that stress can elicit arousal more rapidly from REM sleep compared to NREM sleep, and hypoxia is a stress stimulus caused by airway obstruction in OSA pathophysiology, which may explain why the association in our study was only in OSA patients who were under hypoxic stress but not in the non-OSA group.

Perhaps the association of *FKBP5* variants with sleep disorder was related to the biological role of *FKBP5* in HPA-axis functioning within the neuroendocrine system. *FKBP5* regulates the sensitivity of glucocorticoid receptor (GR) by reducing its cortisol-binding affinity and nuclear translocation, modulating the negative feedback of glucocorticoids to stabilize HPA-axis glucocorticoid output (Dwyer et al., 2020). The HPA axis regulates many vital pathways, including the stress response and sleep regulation, and has been postulated as a possible mechanism for the link between OSA and PTSD/depression (Buckley and Schatzberg, 2005; Gupta and Simpson, 2015; Jaoude et al., 2015). The HPA axis is necessary for maintaining alertness and sleep modulation, and OSA is also correlated with HPA axis dysfunction interactively (Buckley and Schatzberg, 2005). Hyperactivation of the HPA axis can cause more arousals, awakenings and lighter sleep (Nicolaides et al., 2000), which promotes sleep events in OSA, while secondary arousals following intermittent hypoxia episodes can induce HPA activation in turn (Späth-Schwalbe et al., 1991; Buckley and Schatzberg, 2005; Kritikou et al., 2016). Notably, it was also observed that nondepressed offspring with a parental history of depression showed higher cortisol awakening curves, indicating that HPA-axis hyperactivity may partly reflect a genetic susceptibility marker for depression (Vreeburg et al., 2010). In our study, the heritability of HPA activity may be presented by *FKBP5* variations in OSA patients. Previous genetic studies have identified that *FKBP5* polymorphisms rs1360780, rs9470080, and rs9394309 were related to dysfunctional HPA activity (Velders et al., 2011; White et al., 2012; Fani et al., 2013; Pagliaccio et al., 2014). However, it remains unknown whether *FKBP5* variants could regulate sleep and act as a shared genetic factor for the comorbidity by influencing HPA activity or by other biological mechanisms, which merits further biological and clinical investigation. In addition, whether the variants were correlated with other HPA-axis physiological functions, including nutrition metabolism regulation in OSA, was not clear either. Additionally, integrated analysis of eQTL, 3D interacting gene analysis and gene expression patterns revealed that these four SNPs may exert their effects by regulating *FKBP5*, *TULP1*, and *ARMC12*. We found that several genes other than *FKBP5* were also affected by these four SNPs, especially *TULP1* and *ARMC12*, both of which were also detected in neurons involved in sleep regulation. However, there has been no relevant report available on the relationship between the two genes and sleep disorders thus far. Tubby-like protein 1 (*TULP1*) is located approximately 60 kb downstream from *FKBP5* and has been reported to be important for vesicular trafficking of photoreceptor proteins and associated with early-onset retinal degeneration (Remez et al., 2020; Jia et al., 2022). Armadillo repeat containing 12 (*ARMC12*) is located 3 kb upstream of *FKBP5* and has been reported to promote neuroblastoma progression and play crucial roles in spermiogenesis (Li et al., 2018; Shimada et al., 2021). However, the mechanisms underlying the roles of *FKBP5*, *TULP1* and *ARMC12* in regulating sleep need further exploration.

Based on the previous studies presented above, we speculate that *FKBP5* variations increase genetic vulnerability to depression and PTSD, and its potential role in prolonged HPA axis activation may result in a higher hyperarousal state than non-risk allele carriers. Resultant greater sleep fragmentation tends to increase upper airway collapsibility, leading to more sleep breathing events, especially in REM sleep. It is postulated that the two types of diseases may be reciprocal catalysts for each other through possible genetic background overlap, such as *FKBP5* variants. In summary, *FKBP5* variants may connect psychiatric disturbances with OSA and influence OSA phenotypic traits via HPA dysregulation. Additionally, *FKBP5* SNPs might affect sleep breathing via the *FKBP5*, *TULP1*, and *ARMC12* genes.

There were several limitations in this study that should be noted. First, males are predominant in sex distribution among OSA patients, whereas adult women are more likely to develop depression and PTSD than adult men; hence, the sample size of female participants impeded us from analyzing the association comprehensively and in a gender-specific way, and the findings were more generalizable to men than to women. Second, we discussed only the role of the candidate *FKBP5* gene without considering the effect of stress exposure and were unable to evaluate the classic gene by environment interactions in the *FKBP5* gene. Third, we did not consider other complex genetic variations, including indels, DNA methylation and structural variation. The correlation between HPA axis activity and *FKBP5* genotype in OSA patients has not been clarified to acquire a deeper understanding of the connection. Additionally, this study could not provide causative evidence due to its cross-sectional cohort design. Moreover, the study was limited to the Han Chinese population, and further studies are needed to validate whether these genetic variants contribute to OSA risk and related phenotypes in other ethnic groups. Clinical trials and functional experiments are needed to further reveal the mechanism of the *FKBP5* gene and its variation in sleep regulation in the future. The pleiotropic effect of *FKBP5* variants on sleep disorders is worth exploring because of its important biological functions.

## Data availability statement

The original contributions presented in the study are included in the article/Supplementary material, further inquiries can be directed to the corresponding authors.

## Ethics statement

The studies involving human participants were reviewed and approved by the Ethics Committee of Shanghai Sixth People’s Hospital Affiliated to Shanghai Jiao Tong University School of Medicine. The patients/participants provided their written informed consent to participate in this study.

## Author contributions

XL, FL, and SY contributed to the conception and design of the study. XL organized the database. AW and ZW wrote the first draft of the manuscript. AW, ZW, and HY performed the statistical analysis. All authors commented on previous versions of the manuscript and approved the submitted version.

## Funding

The work was supported by STI2030-Major Projects (2021ZD0201900); National Natural Science Foundation of China (82000967, 81971240, 82271153, 82100105); China Postdoctoral Science Foundation (2021M702175); and Shanghai Sixth People’s Hospital (ynts202103).

## Conflict of interest

The research was conducted in the absence of any commercial or financial relationships that could be construed as a potential conflict of interest.

## Publisher’s note

All claims expressed in this article are solely those of the authors and do not necessarily represent those of their affiliated organizations, or those of the publisher, the editors and the reviewers. Any product that may be evaluated in this article, or claim that may be made by its manufacturer, is not guaranteed or endorsed by the publisher.

## Footnotes

^1^ http://asia.ensembl.org/index.html

^2^ https://www.omic.tech/3dsnpv2

^3^ https://human.brain-map.org
