# Supplementary material for: FKBP5 genetic variants are associated with respiratory- and sleep-related parameters in Chinese patients with obstructive sleep apnea
Source: Front Neurosci. 2023 May 18;17:1170889. doi: 10.3389/fnins.2023.1170889 (PMC10233201; doi:10.3389/fnins.2023.1170889)
Supplement: Supplementary file 2 [file Data_Sheet_1.docx]

**Figure S1.** Linkage disequilibrium plots for the SNPs genotyped in the *FKBP5* gene in our study.


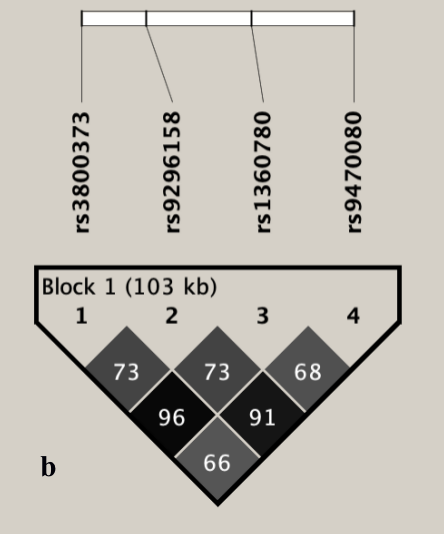

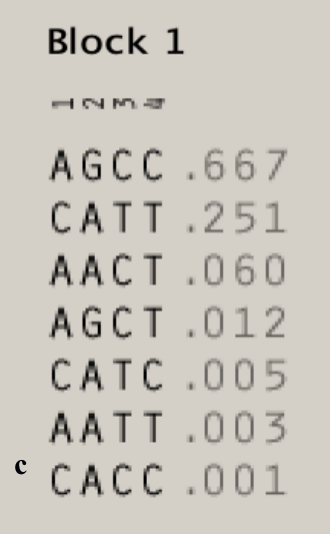

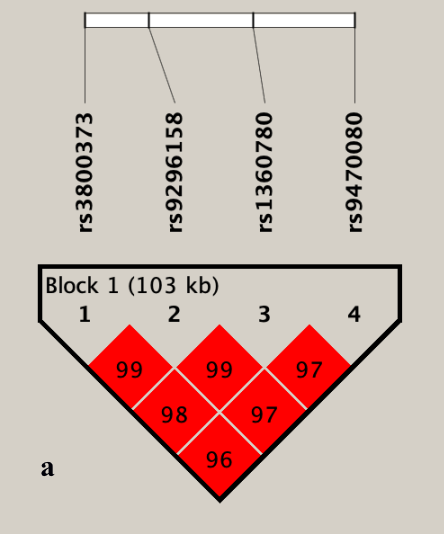


**a.** Shades of red demonstrate the strength of the pairwise linkage disequilibrium based on |D ′|, and the numbers are the values of |D ′| expressed as a percentage.

**b.** Shades of gray demonstrate the strength of the pairwise linkage disequilibrium based on r^2^, and the numbers are the values of r^2^ expressed as a percentage. The haplotype block was defined by the confidence intervals (CI) approach.

**c.** haplotypes and their frequencies were estimated by Haploview.

{participants were divided into 3 subgroups based on previous studies: (i) carriers of at least one risk haplotype, 0 = AGCC/−; (ii) carriers of one risk haplotype and one protective haplotype, 1 = AGCC/CATT; (iii) carriers of at least one protective haplotype, 2 = CATT/−.}

**Figure S2.** Chromosomal locations of four SNPs, *FKBP5* gene and *ARMC12* gene.

**
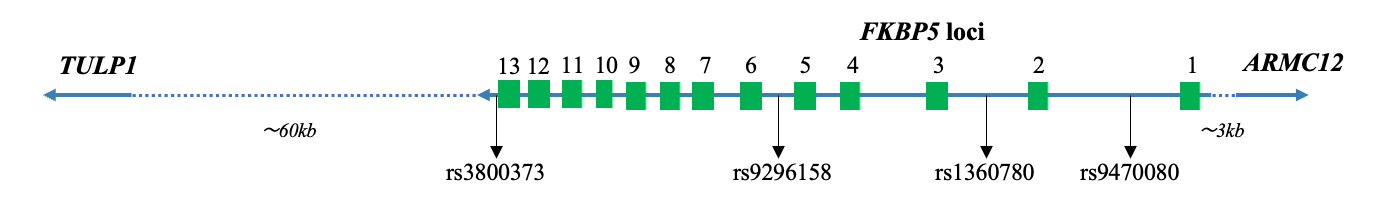
**

Schematic representation of SNPs and nearby *FKBP5, ARMC12* gene. Filled rectangles represent exons of *FKBP5* gene on chromosome 6.

**Figure S3.** Expression of other genes derived from eQTL data in human brains.


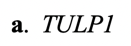

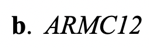

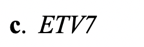

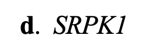

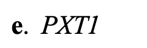

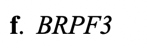


**Table S1.** Associations of the SNPs with clinical characteristics in general population (n=5773).

| Characteristic | rs1360780 | | rs3800373 | | rs9296158 | | rs9470080 | |
| --- | --- | --- | --- | --- | --- | --- | --- | --- |
|  | β | *P* | β | *P* | β | *P* | β | *P* |
| AHI/Total | -0.008 | 0.553 | -0.567 | 0.303 | -0.084 | 0.871 | -0.016 | 0.975 |
| BMI | -0.122 | 0.165 | -0.170 | 0.054 | -0.083 | 0.319 | -0.077 | 0.350 |
| AHI_REM_ | 0.236 | 0.678 | 0.005 | 0.993 | 0.964 | 0.072 | 0.945 | 0.076 |
| AHI_NREM_ | -0.007 | 0.626 | -0.012 | 0.367 | -0.267 | 0.623 | -0.248 | 0.647 |
| MAI | 0.322 | 0.503 | 0.113 | 0.815 | -0.068 | 0.881 | 0.112 | 0.803 |
| WK(SPT) | 0.034 | 0.973 | -0.042 | 0.967 | -0.839 | 0.373 | -0.254 | 0.786 |
| WK/SPT(%) | 0.034 | 0.885 | 0.014 | 0.952 | -0.166 | 0.457 | -0.054 | 0.809 |
| WK(TIB) | 0.725 | 0.631 | 1.077 | 0.478 | -0.093 | 0.947 | 1.163 | 0.409 |
| WK/TIB(%) | 0.090 | 0.77 | 0.173 | 0.578 | -0.045 | 0.877 | 0.139 | 0.629 |
| N1(min) | 0.347 | 0.776 | 0.367 | 0.763 | 0.074 | 0.948 | 0.306 | 0.788 |
| N1/SPT(%) | 0.092 | 0.74 | 0.128 | 0.646 | 0.041 | 0.877 | 0.088 | 0.736 |
| N1/TST(%) | -0.006 | 0.984 | 0.021 | 0.946 | -0.082 | 0.774 | -0.007 | 0.982 |
| N2(min) | 0.402 | 0.813 | 0.131 | 0.939 | 0.686 | 0.669 | -0.784 | 0.623 |
| N2/SPT(%) | -0.069 | 0.85 | -0.179 | 0.628 | 0.003 | 0.994 | -0.230 | 0.504 |
| N2/TST(%) | 0.021 | 0.953 | -0.067 | 0.852 | 0.011 | 0.973 | -0.198 | 0.553 |
| N3(min) | 1.127 | 0.386 | 1.333 | 0.307 | 1.291 | 0.291 | 1.463 | 0.229 |
| N3/SPT(%) | 0.256 | 0.392 | 0.312 | 0.3 | 0.330 | 0.241 | 0.423 | 0.131 |
| N3/TST(%) | 0.210 | 0.502 | 0.288 | 0.36 | 0.227 | 0.44 | 0.319 | 0.277 |
| REM (min) | -0.575 | 0.331 | -0.563 | 0.342 | 0.282 | 0.612 | 0.238 | 0.667 |
| REM/TIB(%) | -0.161 | 0.201 | -0.158 | 0.211 | 0.016 | 0.894 | 0.013 | 0.912 |
| REM/SPT(%) | -0.163 | 0.209 | -0.147 | 0.258 | 0.037 | 0.764 | 0.046 | 0.703 |
| REM/TST(%) | -0.217 | 0.129 | -0.208 | 0.146 | -0.049 | 0.717 | -0.021 | 0.874 |
| TST | 1.298 | 0.436 | 1.223 | 0.464 | 2.306 | 0.141 | 1.181 | 0.449 |
| Sleep efficiency | 0.066 | 0.821 | 0.080 | 0.782 | 0.292 | 0.284 | 0.100 | 0.713 |
| LSpO2 | 0.434 | 0.13 | 0.469 | 0.103 | 0.160 | 0.553 | 0.116 | 0.665 |
| ODI | -0.109 | 0.854 | -0.274 | 0.645 | -0.038 | 0.946 | 0.045 | 0.935 |
| ESS | -0.074 | 0.576 | -0.113 | 0.396 | 0.003 | 0.981 | -0.019 | 0.881 |

**Table S2.** Associations between SNPs and clinical characteristics in non-OSA population (n=744).

| Characteristic | rs1360780 | | rs3800373 | | rs9296158 | | rs9470080 | |
| --- | --- | --- | --- | --- | --- | --- | --- | --- |
|  | β | *P* | β | *P* | β | *P* | β | *P* |
| AHI/Total | -0.146 | 0.083 | -0.151 | 0.07 | -0.141 | 0.077 | -0.164 | 0.042 |
| BMI | 0.043 | 0.844 | 0.002 | 0.986 | 0.052 | 0.802 | 0.086 | 0.661 |
| AHI_REM_ | -0.137 | 0.81 | -0.287 | 0.621 | 0.311 | 0.593 | 0.361 | 0.537 |
| AHI_NREM_ | 0.228 | 0.694 | 0.105 | 0.857 | 0.179 | 0.746 | 0.230 | 0.68 |
| MAI | 0.199 | 0.815 | 0.221 | 0.793 | -0.362 | 0.662 | -0.344 | 0.681 |
| WK(SPT)(min) | 1.209 | 0.705 | 0.919 | 0.776 | 2.022 | 0.496 | 1.948 | 0.517 |
| WK/SPT(%) | 0.594 | 0.444 | 0.526 | 0.497 | 0.726 | 0.324 | 0.691 | 0.353 |
| WK(TIB)(min) | 7.691 | 0.098 | 8.495 | 0.067 | 4.867 | 0.264 | 4.276 | 0.328 |
| WK/TIB(%) | 1.665 | 0.09 | 1.819 | 0.063 | 1.143 | 0.216 | 0.946 | 0.306 |
| N1 (min) | 1.235 | 0.708 | 2.537 | 0.412 | 0.728 | 0.832 | 0.848 | 0.802 |
| N1/SPT(%) | 0.028 | 0.993 | 0.394 | 0.605 | -0.069 | 0.894 | 0.057 | 0.961 |
| N1/TST(%) | 0.510 | 0.541 | 0.792 | 0.33 | 0.116 | 0.9 | 0.047 | 0.972 |
| N2 (min) | -6.819 | 0.118 | -8.282 | 0.055 | -6.161 | 0.136 | -5.919 | 0.156 |
| N2/SPT(%) | -1.761 | 0.061 | -2.143 | 0.021 | -1.773 | 0.047 | -1.502 | 0.095 |
| N2/TST(%) | -1.251 | 0.179 | -1.671 | 0.069 | -1.310 | 0.139 | -1.037 | 0.245 |
| N3 (min) | 2.842 | 0.395 | 3.757 | 0.258 | 4.000 | 0.208 | 2.514 | 0.432 |
| N3/SPT(%) | 0.835 | 0.263 | 1.022 | 0.168 | 1.020 | 0.151 | 0.784 | 0.272 |
| N3/TST(%) | 1.096 | 0.175 | 1.281 | 0.11 | 1.355 | 0.078 | 1.093 | 0.158 |
| REM (min) | -1.554 | 0.342 | -1.821 | 0.26 | -0.890 | 0.573 | -1.047 | 0.51 |
| REM/TIB(%) | -0.325 | 0.373 | -0.383 | 0.288 | -0.193 | 0.583 | -0.203 | 0.568 |
| REM/SPT(%) | -0.314 | 0.421 | -0.359 | 0.352 | -0.203 | 0.59 | -0.189 | 0.62 |
| REM/TST(%) | -0.386 | 0.36 | -0.419 | 0.315 | -0.282 | 0.487 | -0.263 | 0.522 |
| TST | -4.825 | 0.306 | -4.414 | 0.345 | -2.801 | 0.522 | -4.064 | 0.364 |
| Sleep efficiency | -1.160 | 0.199 | -1.075 | 0.231 | -0.644 | 0.441 | -0.811 | 0.342 |
| LSpO2 | 0.125 | 0.705 | 0.168 | 0.606 | 0.175 | 0.576 | 0.140 | 0.66 |
| ODI | 0.776 | 0.372 | 0.702 | 0.419 | 0423 | 0.607 | 0.724 | 0.386 |
| ESS | -0.086 | 0.851 | 0.006 | 0.927 | 0.052 | 0.804 | -0.045 | 0.955 |

**Table S3.** Association of SNPs with sleep-related traits in **women** with moderate OSA (n=249).

| Characteristic | rs1360780 | | rs3800373 | | rs9296158 | | rs9470080 | |
| --- | --- | --- | --- | --- | --- | --- | --- | --- |
|  | β | *P* | β | *P* | β | *P* | β | *P* |
| MAI | 0.008 | 0.996 | -0.431 | 0.814 | -0.513 | 0.767 | -1.038 | 0.533 |
| MAI (4 categories) | -0.009 | 0.929 | -0.049 | 0.646 | -0.068 | 0.501 | -0.055 | 0.569 |
| WK (SPT) (min) | -7.738 | 0.159 | -7.701 | 0.166 | -8.320 | 0.107 | -7.324 | 0.143 |
| WK/SPT(%) | -1.617 | 0.224 | -1.618 | 0.229 | -1.675 | 0.179 | -1.443 | 0.232 |
| WK(TIB)(min) | -1.399 | 0.862 | -0.432 | 0.958 | 1.463 | 0.846 | 0.302 | 0.981 |
| WK(TIB)/TIB(%) | -0.559 | 0.735 | -0.415 | 0.804 | -0.101 | 0.948 | -0.302 | 0.839 |
| Sleep efficiency | 1.379 | 0.368 | 1.517 | 0.327 | 1.555 | 0.278 | 1.338 | 0.336 |
| SE (2 categories) | -0.047 | 0.361 | -0.048 | 0.356 | -0.031 | 0.523 | -0.025 | 0.595 |

**Table** **S4.** Association of the SNPs with the risk of moderate OSA (non and mild OSA vs moderate to severe OSA)

|  | OR | 95% CI | *P* | OR^*^ | 95% CI^*^ | *P^*^* |
| --- | --- | --- | --- | --- | --- | --- |
| rs1360780 | 0.975 | (0.876-1.085) | 0.640 | 0.978 | (0.877-1.090) | 0.683 |
| rs3800373 | 0.970 | (0.872-1.080) | 0.583 | 0.968 | (0.868-1.079) | 0.556 |
| rs9296158 | 0.979 | (0.885-1.083) | 0.685 | 0.983 | (0.887-1.089) | 0.738 |
| rs9470080 | 0.991 | (0.896-1.095) | 0.854 | 0.993 | (0.897-1.100) | 0.900 |
| haplotype | 0.978 | (0.886-1.080) | 0.659 | 0.976 | (0.883-1.079) | 0.639 |

**Table S5**. Interacting genes of SNPs in 3D chromatin loop.

| a.rs1360780 interacting genes via chromatin loop | | | | | | |
| --- | --- | --- | --- | --- | --- | --- |
| Gene | Loop type | Loop start | Loop end | Distance | Cell type | Tissue |
| ARMC12 | Within loop | chr6:35537777-35547777 | chr6:35697777-35707777 | 160000 | Astrocyte |  |
| ARMC12 | Within loop | chr6:35587777-35597777 | chr6:35697777-35707777 | 110000 | Astrocyte |  |
| ARMC12 | Within loop | chr6:35547777-35557777 | chr6:35707777-35717777 | 160000 | Cortex_DLPFC | Cortex_DLPFC |
| CLPS | Within loop | chr6:35587777-35597777 | chr6:35757777-35767777 | 170000 | Pancreas | Pancreas |
| CLPSL1 | Within loop | chr6:35567777-35577777 | chr6:35747777-35757777 | 180000 | Spleen | Spleen |
| CLPSL2 | Within loop | chr6:35567777-35577777 | chr6:35747777-35757777 | 180000 | Spleen | Spleen |
| FKBP5 | Within loop | chr6:35537777-35547777 | chr6:35697777-35707777 | 160000 | Astrocyte |  |
| FKBP5 | Within loop | chr6:35587777-35597777 | chr6:35697777-35707777 | 110000 | Astrocyte |  |
| FKBP5 | Within loop | chr6:35547777-35557777 | chr6:35707777-35717777 | 160000 | Cortex_DLPFC | Cortex_DLPFC |
| FKBP5 | Within loop | chr6:35527777-35537777 | chr6:35657777-35667777 | 130000 | Hippocampus | Hippocampus |
| FKBP5 | Within loop | chr6:35527777-35537777 | chr6:35657777-35667777 | 130000 | Astrocyte |  |
| LOC285847 | Within loop | chr6:35537777-35547777 | chr6:35697777-35707777 | 160000 | Astrocyte |  |
| LOC285847 | Within loop | chr6:35587777-35597777 | chr6:35697777-35707777 | 110000 | Astrocyte |  |
| LOC285847 | Within loop | chr6:35547777-35557777 | chr6:35707777-35717777 | 160000 | Cortex_DLPFC | Cortex_DLPFC |
| MIR5690 | Within loop | chr6:35537777-35547777 | chr6:35697777-35707777 | 160000 | Astrocyte |  |
| MIR5690 | Within loop | chr6:35587777-35597777 | chr6:35697777-35707777 | 110000 | Astrocyte |  |
| MIR5690 | Within loop | chr6:35547777-35557777 | chr6:35707777-35717777 | 160000 | Cortex_DLPFC | Cortex_DLPFC |
| MIR5690 | Within loop | chr6:35527777-35537777 | chr6:35657777-35667777 | 130000 | Hippocampus | Hippocampus |
| MIR5690 | Within loop | chr6:35527777-35537777 | chr6:35657777-35667777 | 130000 | Astrocyte |  |
| TULP1 | Within loop | chr6:35477777-35487777 | chr6:35627777-35637777 | 150000 | HAP1_SCC4_KO | HAP1_SCC4_KO |
| b.rs3800373 interacting genes via chromatin loop | | | | | | |
| Gene | Loop type | Loop start | Loop end | Distance | Cell type | Tissue |
| ARMC12 | Within loop | chr6:35537777-35547777 | chr6:35697777-35707777 | 160000 | Astrocyte |  |
| DEF6 | Anchor to anchor | chr6:35257777-35267777 | chr6:35537777-35547777 | 280000 | HMEC | Breast |
| FKBP5 | Within loop | chr6:35537777-35547777 | chr6:35697777-35707777 | 160000 | Astrocyte |  |
| FKBP5 | Within loop | chr6:35507777-35517777 | chr6:35677777-35687777 | 170000 | H1-NPC |  |
| FKBP5 | Within loop | chr6:35527777-35537777 | chr6:35657777-35667777 | 130000 | Hippocampus | Hippocampus |
| FKBP5 | Within loop | chr6:35527777-35537777 | chr6:35657777-35667777 | 130000 | Astrocyte |  |
| LHFPL5 | Anchor to anchor | chr6:35537777-35547777 | chr6:35767777-35777777 | 230000 | Bowel_Small |  |
| LOC285847 | Within loop | chr6:35537777-35547777 | chr6:35697777-35707777 | 160000 | Astrocyte |  |
| MIR5690 | Within loop | chr6:35537777-35547777 | chr6:35697777-35707777 | 160000 | Astrocyte |  |
| MIR5690 | Within loop | chr6:35527777-35537777 | chr6:35657777-35667777 | 130000 | Hippocampus | Hippocampus |
| MIR5690 | Within loop | chr6:35527777-35537777 | chr6:35657777-35667777 | 130000 | Astrocyte |  |
| TULP1 | Within loop | chr6:35477777-35487777 | chr6:35627777-35637777 | 150000 | HAP1_SCC4_KO |  |
| c.rs9470080 interacting genes via chromatin loop | | | | | | |
| Gene | Loop type | Loop start | Loop end | Distance | Cell type | Tissue |
| ARMC12 | Within loop | chr6:35537777-35547777 | chr6:35697777-35707777 | 160000 | Astrocyte |  |
| ARMC12 | Within loop | chr6:35587777-35597777 | chr6:35697777-35707777 | 110000 | Astrocyte |  |
| ARMC12 | Within loop | chr6:35547777-35557777 | chr6:35707777-35717777 | 160000 | Cortex_DLPFC | DLPFC |
| CLPS | Within loop | chr6:35587777-35597777 | chr6:35757777-35767777 | 170000 | Pancreas | Pancreas |
| CLPSL1 | Within loop | chr6:35567777-35577777 | chr6:35747777-35757777 | 180000 | Spleen | Spleen |
| CLPSL2 | Within loop | chr6:35567777-35577777 | chr6:35747777-35757777 | 180000 | Spleen | Spleen |
| FKBP5 | Within loop | chr6:35537777-35547777 | chr6:35697777-35707777 | 160000 | Astrocyte |  |
| FKBP5 | Within loop | chr6:35587777-35597777 | chr6:35697777-35707777 | 110000 | Astrocyte |  |
| FKBP5 | Within loop | chr6:35547777-35557777 | chr6:35707777-35717777 | 160000 | Cortex_DLPFC | DLPFC |
| FKBP5 | Within loop | chr6:35527777-35537777 | chr6:35657777-35667777 | 130000 | Hippocampus | Hippocampus |
| FKBP5 | Within loop | chr6:35527777-35537777 | chr6:35657777-35667777 | 130000 | Astrocyte |  |
| LOC285847 | Within loop | chr6:35537777-35547777 | chr6:35697777-35707777 | 160000 | Astrocyte |  |
| LOC285847 | Within loop | chr6:35587777-35597777 | chr6:35697777-35707777 | 110000 | Astrocyte |  |
| LOC285847 | Within loop | chr6:35547777-35557777 | chr6:35707777-35717777 | 160000 | Cortex_DLPFC | DLPFC |
| MIR5690 | Within loop | chr6:35537777-35547777 | chr6:35697777-35707777 | 160000 | Astrocyte |  |
| MIR5690 | Within loop | chr6:35587777-35597777 | chr6:35697777-35707777 | 110000 | Astrocyte |  |
| MIR5690 | Within loop | chr6:35547777-35557777 | chr6:35707777-35717777 | 160000 | Cortex_DLPFC | DLPFC |
| MIR5690 | Within loop | chr6:35527777-35537777 | chr6:35657777-35667777 | 130000 | Hippocampus | Hippocampus |
| MIR5690 | Within loop | chr6:35527777-35537777 | chr6:35657777-35667777 | 130000 | Astrocyte |  |
| d.rs9296158 interacting genes via chromatin loop | | | | | | |
| Gene | Loop type | Loop start | Loop end | Distance | Cell type | Tissue |
| ARMC12 | Within loop | chr6:35537777-35547777 | chr6:35697777-35707777 | 160000 | Astrocyte |  |
| ARMC12 | Within loop | chr6:35547777-35557777 | chr6:35707777-35717777 | 160000 | Cortex_DLPFC | DLPFC |
| CLPSL1 | Within loop | chr6:35547777-35557777 | chr6:35747777-35757777 | 200000 | RPMI7951 |  |
| CLPSL2 | Within loop | chr6:35547777-35557777 | chr6:35747777-35757777 | 200000 | RPMI7951 |  |
| CLPSL2 | Within loop | chr6:35557777-35567777 | chr6:35737777-35747777 | 180000 | PANC1 |  |
| FKBP5 | Within loop | chr6:35537777-35547777 | chr6:35697777-35707777 | 160000 | Astrocyte |  |
| FKBP5 | Within loop | chr6:35547777-35557777 | chr6:35707777-35717777 | 160000 | Cortex_DLPFC |  |
| FKBP5 | Within loop | chr6:35527777-35537777 | chr6:35657777-35667777 | 130000 | Hippocampus | Hippocampus |
| FKBP5 | Within loop | chr6:35527777-35537777 | chr6:35657777-35667777 | 130000 | Astrocyte |  |
| LOC285847 | Within loop | chr6:35537777-35547777 | chr6:35697777-35707777 | 160000 | Astrocyte |  |
| LOC285847 | Within loop | chr6:35547777-35557777 | chr6:35707777-35717777 | 160000 | Cortex_DLPFC | DLPFC |
| MIR5690 | Within loop | chr6:35537777-35547777 | chr6:35697777-35707777 | 160000 | Astrocyte |  |
| MIR5690 | Within loop | chr6:35547777-35557777 | chr6:35707777-35717777 | 160000 | Cortex_DLPFC | DLPFC |
| MIR5690 | Within loop | chr6:35527777-35537777 | chr6:35657777-35667777 | 130000 | Hippocampus | Hippocampus |
| MIR5690 | Within loop | chr6:35527777-35537777 | chr6:35657777-35667777 | 130000 | Astrocyte |  |
| TULP1 | Within loop | chr6:35477777-35487777 | chr6:35627777-35637777 | 150000 | HAP1_SCC4_KO |  |

**Table S6.** Gene expression correlations of four *FKBP5* SNPs from Ensembl data

Presented in additional Excel file (Supplementary_TableS9.xlsx)

**Table S7.** Gene expression correlations of four SNPs with *FKBP5* gene from Ensembl data

| a. gene expression correlations of rs1360780 | | | | | |
| --- | --- | --- | --- | --- | --- |
| Gene Symbol | Ensembl ID | P-value (-log10) | Effect size | Tissue | P-value |
| FKBP5 | ENSG00000096060 | 2.31185704 | -0.0568768 | Cells_Cultured_fibroblasts | 0.00487689 |
| FKBP5 | ENSG00000096060 | 2.094115651 | -0.039878 | monocyte_IAV | 0.00805164 |
| FKBP5 | ENSG00000096060 | 1.999427109 | 0.0571667 | iPSC | 0.0100132 |
| FKBP5 | ENSG00000096060 | 1.887492189 | -0.0513107 | monocyte_R848 | 0.0129571 |
| FKBP5 | ENSG00000096060 | 1.811724151 | -0.0414014 | Muscle_Skeletal | 0.0154268 |
| FKBP5 | ENSG00000096060 | 1.515165098 | 0.109377 | Liver | 0.0305376 |
| FKBP5 | ENSG00000096060 | 1.510033088 | -0.0432599 | Skin_Sun_Exposed_Lower_leg | 0.0309006 |
| FKBP5 | ENSG00000096060 | 1.434207652 | 0.0781346 | LCL | 0.0367953 |
| FKBP5 | ENSG00000096060 | 1.431784191 | 0.109768 | Brain_Frontal_Cortex_BA9 | 0.0370012 |
| b. gene expression correlations of rs3800373 | | | | | |
| Gene Symbol | Ensembl ID | P-value (-log10) | Effect size | Tissue | P-value |
| FKBP5 | ENSG00000096060 | 2.810352504 | -0.0646584 | Cells_Cultured_fibroblasts | 0.00154756 |
| FKBP5 | ENSG00000096060 | 2.598961522 | -0.0526184 | Muscle_Skeletal | 0.0025179 |
| FKBP5 | ENSG00000096060 | 1.821230183 | 0.130941 | Brain_Frontal_Cortex_BA9 | 0.0150928 |
| FKBP5 | ENSG00000096060 | 1.707588851 | 0.0519696 | iPSC | 0.019607 |
| FKBP5 | ENSG00000096060 | 1.638733138 | 0.0894686 | LCL | 0.0229756 |
| FKBP5 | ENSG00000096060 | 1.523249501 | 0.113435 | Liver | 0.0299744 |
| FKBP5 | ENSG00000096060 | 1.49883252 | 0.208513 | sensory_neuron | 0.0317079 |
| FKBP5 | ENSG00000096060 | 1.480496208 | -0.0317206 | monocyte_IAV | 0.0330753 |
| FKBP5 | ENSG00000096060 | 1.456352805 | -0.0436236 | Heart_Left_Ventricle | 0.0349661 |
| FKBP5 | ENSG00000096060 | 1.446506557 | -0.0424062 | Skin_Sun_Exposed_Lower_leg | 0.0357679 |
| FKBP5 | ENSG00000096060 | 1.375715841 | -0.0414516 | monocyte_R848 | 0.0421002 |
| c. gene expression correlations of rs9470080 | | | | | |
| Gene Symbol | Ensembl ID | P-value (-log10) | Effect size | Tissue | P-value |
| FKBP5 | ENSG00000096060 | 2.402779946 | -0.0557442 | Cells_Cultured_fibroblasts | 0.00395567 |
| FKBP5 | ENSG00000096060 | 2.349965576 | 0.0604974 | iPSC | 0.00446719 |
| FKBP5 | ENSG00000096060 | 2.312916263 | 0.150028 | Brain_Frontal_Cortex_BA9 | 0.00486501 |
| FKBP5 | ENSG00000096060 | 2.036721823 | -0.0428651 | Muscle_Skeletal | 0.00918921 |
| FKBP5 | ENSG00000096060 | 1.919373513 | 0.120309 | Liver | 0.01204 |
| FKBP5 | ENSG00000096060 | 1.89618602 | -0.0372487 | monocyte_IAV | 0.0127003 |
| FKBP5 | ENSG00000096060 | 1.717572763 | 0.0901574 | LCL | 0.0191614 |
| FKBP5 | ENSG00000096060 | 1.713795108 | 0.0348885 | Whole_Blood | 0.0193288 |
| FKBP5 | ENSG00000096060 | 1.650056482 | 0.0806687 | brain_naive | 0.0223843 |
| FKBP5 | ENSG00000096060 | 1.643771392 | 0.0559335 | CD4_T-cell_anti-CD3-CD28 | 0.0227106 |
| FKBP5 | ENSG00000096060 | 1.31348796 | 0.187354 | sensory_neuron | 0.0485861 |
| d. gene expression correlations of rs9296158 | | | | | |
| Gene Symbol | Ensembl ID | P-value (-log10) | Effect size | Tissue | P-value |
| FKBP5 | ENSG00000096060 | 2.260146128 | -0.0552056 | Cells_Cultured_fibroblasts | 0.00549356 |
| FKBP5 | ENSG00000096060 | 1.978922391 | 0.0619483 | CD4_T-cell_anti-CD3-CD28 | 0.0104973 |
| FKBP5 | ENSG00000096060 | 1.952818623 | -0.0427814 | Muscle_Skeletal | 0.0111476 |
| FKBP5 | ENSG00000096060 | 1.828604145 | 0.0536658 | iPSC | 0.0148387 |
| FKBP5 | ENSG00000096060 | 1.777718041 | 0.126388 | Brain_Frontal_Cortex_BA9 | 0.0166833 |
| FKBP5 | ENSG00000096060 | 1.624791948 | 0.114369 | Liver | 0.0237251 |
| FKBP5 | ENSG00000096060 | 1.536339891 | -0.0432596 | Skin_Sun_Exposed_Lower_leg | 0.0290844 |
| FKBP5 | ENSG00000096060 | 1.443572055 | 0.0791077 | LCL | 0.0360104 |
| FKBP5 | ENSG00000096060 | 1.396124116 | -0.0303192 | monocyte_IAV | 0.0401676 |
| FKBP5 | ENSG00000096060 | 1.338438123 | -0.040987 | monocyte_R848 | 0.0458735 |
